# Taxogenomic Analysis of a Novel Yeast Species, *Lachancea rosae* Sp. Nov. F.A., Isolated From the Wild Rose *Rosa californica*

**DOI:** 10.1002/yea.70000

**Published:** 2025-09-01

**Authors:** Yakendra Bajgain, Quinn K. Langdon, Cara M. Krien, Martin Jarzyna, Kelly V. Buh, Max A. B. Haase, Anthony Pasles, John F. Wolters, Marizeth Groenewald, Chris Todd Hittinger, Dana A. Opulente

**Affiliations:** ^1^ Biology Department Villanova University Villanova Pennsylvania USA; ^2^ Laboratory of Genetics, Wisconsin Energy Institute, Center for Genomic Science Innovation, J. F. Crow Institute for the Study of Evolution University of Wisconsin–Madison Madison Wisconsin USA; ^3^ DOE Great Lakes Bioenergy Research Center University of Wisconsin–Madison Madison Wisconsin USA; ^4^ Department of Mechanistic Cell Biology Max Planck Institute of Molecular Physiology Dortmund Germany; ^5^ Department of Biology New York University New York New York USA; ^6^ Westerdijk Fungal Biodiversity Institute Utrecht the Netherlands

## Abstract

A novel *Saccharomycotina* yeast strain, yHQL494, was isolated from the rose hip of the wild rose *Rosa californica* from Castle Crags State Park, California, USA. Phylogenetic analyses of both whole genome data and the sequences from the D1/D2 region of the large ribosomal subunit (LSU) rRNA gene placed strain yHQL494 within the genus *Lachancea* and grouped it into a clade with *Lachancea lanzarotensis* and *Lachancea meyersii*. Taxogenomic analyses were conducted on publicly available genome sequences to gain a deeper insight into the carbon and nitrogen gene‐trait associations across the *Lachancea* clade. The results of these analyses were found to be consistent across *Lachancea* species. Growth assays and microscopic analyses were conducted to determine the physiological characteristics of strain yHQL494, including the presence of hyphae or pseudohyphae, ascospore formation, fermentation abilities, and assimilation of carbon and nitrogen compounds. Based on the phenotypic and genomic characteristics of the strain yHQL494^T^ (=NRRL Y‐64858^T^, =CBS 18,574^T^), we propose a new species, *Lachancea rosae* sp. nov. f.a.

## Summary

- The novel species *Lachancea rosae* is discovered and formally described.
- Taxogenomic analysis shows how carbon and nitrogen gene presence correlates with traditional physiological assays.

## Graphical abstract

The discovery, description, and taxogenomic analysis of a novel species of *Lachancea* revealed correlations between gene content and carbon and nitrogen physiological assays.

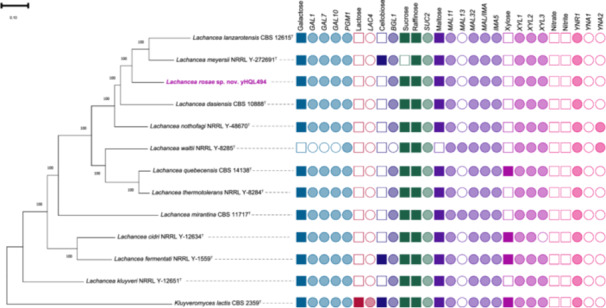

## 1 Introduction

The genus *Lachancea* belongs to the order *Saccharomycetales* and family *Saccharomycetaceae* (Groenewald et al. 2023). It was first proposed by Dr. Cletus P. Kurtzman in 2003 and was named in honor of Prof. Marc‐André Lachance. Currently, the genus consists of 11 described species (Kurtzman 2003; Porter et al. 2019; Opulente et al. 2024). The first five species described were previously classified in other genera, sometimes multiple genera, and were reassigned to *Lachancea* as *Lachancea fermentati* (previously *Zygosaccharomyces fermentati*), *Lachancea cidri* (previously *Zygosaccharomyces cidri*), *Lachancea thermotolerans* (previously *Zygosaccharomyces thermotolerans*), *Lachancea kluyveri* (previously *Saccharomyces kluyveri*), and *Lachancea waltii* (previously *Kluyveromyces waltii*), with *L. thermotolerans* as the type species for the genus (Kurtzman 2003; Porter et al. 2019; Opulente et al. 2024). Since the circumscription of *Lachancea* (Kurtzman 2003), six additional species have been formally described, *Lachancea meyersii*, *Lachancea dasiensis*, *Lachancea nothofagi*, *Lachancea mirantina*, *Lachancea lanzarotensis*, and *Lachancea quebecensis* (Fell et al. 2004; Lee et al. 2009; Mestre et al. 2010; Pereira et al. 2011; González et al. 2013; Freel et al. 2015).

*Lachancea* spp. have been isolated from multiple ecological niches, including plants, soil, food products, and beverages (Kurtzman et al. 2011; Opulente et al. 2018, 2024; Porter et al. 2019; Spurley et al. 2022). With a recent focus in exploring the enological importance of non‐*Saccharomyces* yeast spp., *L. fermentati* (Bellut et al. 2020), *L. thermotolerans* (Jolly et al. 2014), and *L. lanzarotensis have* been shown to be economically important due to their fermentative capabilities. In particular, *L. thermotolerans* has been shown to influence the aroma and flavor of wine through the production of esters, terpenes, and 3‐methylthio‐1‐propanol (Jolly et al. 2014; Benito 2018). Even though members of the genus *Lachancea* have not been implicated in any fungal diseases, there was a case where *L. fermentati* was attributed to causing fungemia in an immunocompromised individual (Leuck et al. 2014). However, such cases are rare, and *Lachancea* species are mostly associated with fermentation processes.

Using previously established yeast enrichment and isolation protocols (Sylvester et al. 2015; Opulente et al. 2019; Spurley et al. 2022), we identified a candidate for a novel *Lachancea* species, represented by strain yHQL494, that was isolated from the rose hips of the wild rose *Rosa californica* in Castle Crags State Park, California. Strain yHQL494 was suggested to represent a novel species after the internal transcribed spacer region (ITS) and the D1/D2 region of the large ribosomal subunit (LSU) rRNA gene were sequenced and compared to publicly available sequences (Kurtzman and Robnett 1998, 2013). In addition to traditional taxonomic methods, we performed whole genome sequencing and gene presence analyses to provide further insight into its genetic makeup, growth patterns, and phylogenetic relationship with known *Lachancea* species.

## 2 Materials and Methods

### 2.1 Species Isolation and Identification

Established enrichment protocols were used to isolate strain yHQL494 from a rose hip sample (Sylvester et al. 2015; Opulente et al. 2019; Spurley et al. 2022). The sample was collected by a community scientist, Bill Vagt, from the wild rose *R. californica* in Castle Crags State Park, California (41°9'9.825“N X 122°18'40.743“W), and the strain was isolated and identified by Quinn K. Langdon (Table S1). The sample was incubated at 30०C in liquid synthetic complete (SC) medium (yeast nitrogen base without amino acids, ammonium sulfate, or glucose 6.7 g/L; ammonium sulfate 5 g/L; drop‐out mix 1.7 g/L) with 8% glucose. Once there was visible growth, a secondary enrichment step was conducted in fresh SC medium with 8% glucose at 30०C before the cultures were plated on a yeast extract‐peptone‐dextrose‐agar (YPDA—yeast extract 10 g/L, peptone 20 g/L, glucose 20 g/L, agar 20 g/L) and also incubated at 30°C. Unique colony morphologies were sequenced using Sanger sequencing of the ITS and the D1/D2 region of the LSU rRNA gene for preliminary species identification.

## 3 Morphological and Physiological Characterization

Characteristics of the novel species were described through microscopic examination, as well as growth tests in liquid and solid media. Standard methods proposed by Kurtzman et al. (2011) were used to determine the physiological characteristics(Kurtzman et al. 2011). Briefly, to test the ability of the strain to assimilate specific carbon sources, a colony of strain yHQL494 was pre‐cultured in 0.1% glucose minimal medium (MM—yeast nitrogen base without amino acids, ammonium sulfate, or glucose 6.7 g/L; ammonium sulfate 5 g/L; 1 g/L glucose) and grown at 22°C ± 1°C for 1 day. The resulting culture was inoculated into liquid media containing different carbon sources, each at a concentration of 1%, and growth was visually inspected for 4 weeks at 22°C ± 1°C (Kurtzman et al. 2011). To test the ability of the strain to assimilate specific nitrogen sources, a colony of strain yHQL494 was pre‐cultured in YPD medium and grown at 22°C ± 1°C for 1 day. The resulting culture was inoculated into liquid minimal media (MM—yeast nitrogen base without amino acids, ammonium sulfate, or glucose 6.7 g/L; 10 g/L glucose) containing different nitrogen sources and grown for 1 week (Kurtzman et al. 2011). Cultures were then inoculated into fresh media for each nitrogen source, and growth was visually inspected for 4 weeks at 22°C ± 1°C. The Dalmau Plate method was used to determine the formation of true hyphae or pseudohyphae on YPDA plates. The colony morphology was determined using plate cultures grown on 2% Glucose, 0.5% yeast extract, 1% peptone, and 1.5% agar (GYPA) for 7 days at 25°C. Additionally, the strain was grown on GYPA, YPDA, malt‐extract agar (MEA), V8‐juice agar (V8), yeast extract‐malt extract agar (YMA), and yeast carbon base with ammonium sulfate (YCBAS) agar plates and incubated at 25°C for up to 2 months at 10°C and 25°C, and the plates were examined every week under a microscope to determine the formation of ascospores.

## 4 Taxogenomic Analyses

### 4.1 Genome Assembly

Whole genome sequence analyses were performed using the publicly available genome sequence JAJMGS000000000 and short read data SRR16988996.

### 4.2 Phylogenetic Analyses

The bioinformatic pipeline *HybPiper* (Johnson et al. 2016) was used to isolate and assemble the raw paired‐end reads of the D1/D2 region of the LSU rRNA gene and ITS region for strain yHQL494 using D1/D2 and ITS sequences from *Saccharomyces cerevisiae*. GenBank numbers for the ITS and the D1/D2 region of the LSU rRNA gene sequences for strain yHQL494 are ON040667 and ON064074, respectively. Sequences that have been extracted from genomes using bioinformatic tools such as *HybPiper* can be verified by using alignment tools such as BLAST to ensure accuracy. In the case of barcode sequences, you can align to closely related species. YeastIP, a database that contains the multiple barcode sequences of many species of yeasts in the subphylum *Saccharomycotina* (Weiss et al. 2013), along with GenBank and the Westerdijk Fungal Biodiversity Institute (CBS database), were used to obtain the publicly available *Lachancea* ITS and D1/D2 sequences (Table S2). These sequences were used to determine the relatedness of strain yHQL494 with known *Lachancea* species, phylogenies were made for the ITS region and D1/D2 region of the LSU rRNA gene of known *Lachancea* species and strains in MEGA (Kumar et al. 2018). The default parameters for CLUSTALW were used to align those sequences and a neighbor‐joining phylogeny was constructed using the default settings in MEGA with 1000 bootstrap replicates.

Pairwise ANI values were calculated from publicly available genomes using *fastANI*() with fragLen set to 500 bp (Opulente et al. 2024). The R package *pheatmap* (v. 1.0.12) was used to visualize the results of the ANI analysis. The ANI values were converted to a distanced by subtracting 100 from each value and then scaled using the scale parameter available in the function. Hierarchical clustering was performed using the average method.

### 4.3 Gene Trait Analyses

The methods and sequences described in Opulente et al. (2018) were used to detect gene presence for all publicly available genomes from *Lachancea* species and strain yHQL494. Most of the gene sequences are from *S. cerevisiae* (Opulente et al. 2018). However, the sequence for *LAC4* was from *Kluyveromyces lactis*, *XYL1 and XYL3* was from *Schefferomyces stipitis*, and YNR1, YNA1, and YNA2 were from *Ogataea polymorpha* (Opulente et al. 2018). The BLAST algorithm was used (tblastx), and an *e*‐value cutoff of 10^−10^ was used to infer gene presence (Altschul et al. 1990; Opulente et al. 2023). Genes that are known paralogs were combined and only reported once (Voordeckers et al. 2012; Opulente et al. 2018).

## 5 Results and Discussions

### 5.1 Phylogenetic Analyses

Initial BLAST alignments were used to assess sequence similarity between strain yHQL494 and the type strains of other closely related *Lachancea* species. For the 524‐nt D1/D2 region of the LSU rRNA gene, strain yHQL494 showed the highest sequence with *L. dasiensis* (98.04%; 9 substitutions, and 1 indel), followed by *L. nothofagi* (97.54%; 11 substitutions, 2 indels), *L. meyersii* (97.05%; 11 substitution, 5 indels), and *L. lanzarotensis* (96.60%; 15 substitutions, 3 indels) (Table S3). In the 604‐nt ITS region, strain yHQL494 shared 96.01% identity with *L. meyersii* (21 substitutions, 6 indels), 95.67% with *L. nothofagi* (24 substitutions, 2 indels), 94.67% with *L. dasiensis* (28 substitutions, 4 indels), and 94.52% identity with *L. lanzarotensis* (27 substitutions, 6 indels) (Table S3).

**Figure 1:**
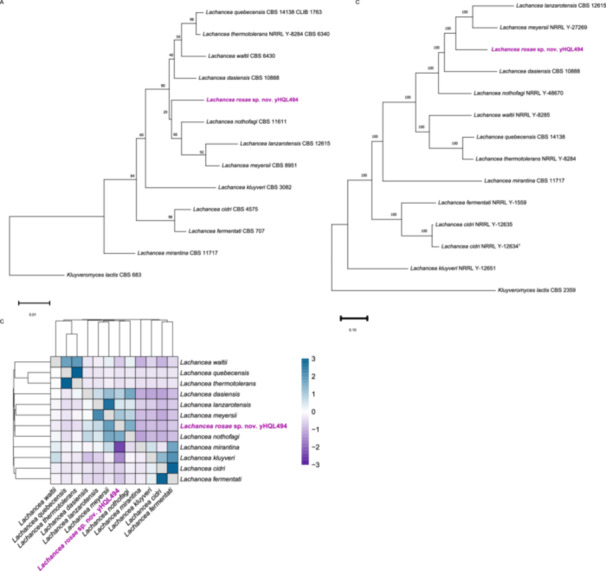
(A) Neighbor‐Joining tree for the taxonomic type strains of the 11 described *Lachancea* species and *Lachancea rosae* sp. nov. (yHQL494) based on the D1/D2 region of the LSU rRNA gene. Bootstrap values (*n* = 1000) are indicated by numbers at the nodes. (B) Whole genome phylogeny pruned from (Opulente et al. 2024). Bootstrap values are indicated by numbers at the nodes. (C) Heatmap and hierarchical cluster analysis of pairwise average nucleotide index (ANI) values. Blue boxes indicate species that have higher ANI values, while purple boxes indicate lower ANI values. Hierarchical clustering was performed using the average method.

A phylogenetic analysis based on the D1/D2 region of the LSU rRNA gene placed strain yHQL494 in a clade with *L. meyersii*, *L. lanzarotensis*, and *L. nothofagi* (Figure 1A). Given the discrepancies between the BLAST results and the D1/D2 phylogeny, we extended our analysis to include additional *Lachancea* strains for all species in the genus for both the ITS region and D/D2 region (Figure S1A‐B, Table S4). In both our expanded ribosomal gene trees, strain yHQL494 was consistently in a clade with *L. meyersii*, *L. lanzarotensis*, and *L. nothofagi*.

We compared our D1/D2 phylogeny (Figure 1A) with a pruned genome‐scale phylogenetic tree (Figure 1B) and found a strong topological similarity, with strain yHQL494 again in a clade with *L. meyersii* and *L. lanzarotensis* (Opulente et al. 2024). To (Figure 1C, Table S4). To further assess genomic relatedness, we calculated pairwise average nucleotide identity (ANI) values among *Lachancea* type strains. Hierarchical clustering of ANI values revealed species‐level structure, with strain yHQL494 sharing its highest ANI (75.72%) with *L. meyersii* (Figure 1C, Table S5).

### 5.2 Genomic and Phenotypic Analyses

**Figure 2:**
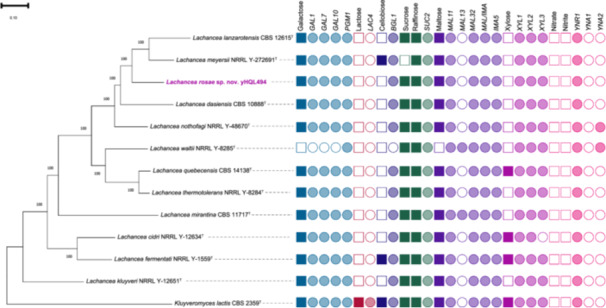
Metabolic traits and underlying genes for the carbon sources galactose, lactose, cellobiose, sucrose, raffinose, maltose, and xylose, as well as for the nitrogen sources nitrate and nitrite. Filled in squares indicates the ability to utilize a carbon or nitrogen source. Filled in circles correspond to gene presence. Gene presence was determined by BLAST analyses.

The steady incorporation of whole genome sequences in the identification and taxonomic classification of novel yeast and bacterial species has delineated previously unknown genotype‐phenotype associations and helped construct robust phylogenies (Shen et al. 2018; Čadež et al. 2021; Opulente et al. 2024). We determined gene presence across *Lachancea* genomes to associate genome content with the utilization capacity of the yeasts on the different carbon and nitrogen sources that were tested (Figure 2). Our analyses contained multiple genes implicated in the metabolism of various carbon and nitrogen sources across yeasts (Table S6). In many cases, there was good correspondence in the genotype‐phenotype map (Figure 2). For example, all species except *L. waltii* possess *GAL1, GAL7, and GAL10* and are capable of metabolizing galactose. The inability of *L. waltii* to grow on galactose is consistent with previous studies (Haase et al. 2021). Below we focused on the exception to the genotype‐phenotype map.

The absence of *YNA1* and *YNA2* from the genome of the majority of *Lachancea* species corroborated their inability to assimilate nitrite and nitrate compounds. Both *YNA1* and *YNA2* encode transcription factors involved in regulating nitrate and nitrite metabolism (Shen et al. 2018; Opulente et al. 2023). These genes were a more reliable indicator of this metabolism than homologs of the reductases encoded by *YNR1*, which was present in all genomes, a phenomenon previously noted (Opulente et al. 2023). Consistent with other literature (Nalabothu et al. 2023), the presence of xylose metabolism genes (*XYL1*, *XYL2*, and *XYL3*) did not always predict growth on xylose; 60% (9/15) of the *Lachancea* species possessed the genes necessary for growth on xylose but were unable to assimilate it (Figure 2, Table S6). Conversely, we observed that approximately 18% (3/15) of *Lachancea* species were able to utilize cellobiose; however, we were able to detect multiple *BGL1* homologs, which encode β‐glucosidases associated with the utilization of cellobiose in some yeasts (Gurgu et al. 2011; Shen et al. 2018; Opulente et al. 2023), in all *Lachancea* genomes. This discrepancy suggests that not all *BGL1* homologs are capable of supporting growth on cellobiose. Additionally, *L. waltii* lacks the ability to grow on maltose, despite containing homologs of all of the genes required for its metabolism (*MAL11*, *MAL12*, *MAL13*, *IMA1‐4*, and *IMA5*). This inability to grow in a certain medium despite containing the genes required for the metabolism of carbon sources in that medium has been attributed to the factors beyond gene presence or absence, including patterns of gene expression regulation and enzyme specificity (Riley et al. 2016).

## 6 Description of *L. rosae* F.A., Sp. Nov

Y. Bajgain, Q.K. Langdon, C.M. Krien, M. Jarzyna, K.V. Buh, M.A.B. Haase, A. Pasles, M. Groenew., Hittinger, D.A. Opulente. Mycobank no. MB857814.

**Figure 3:**
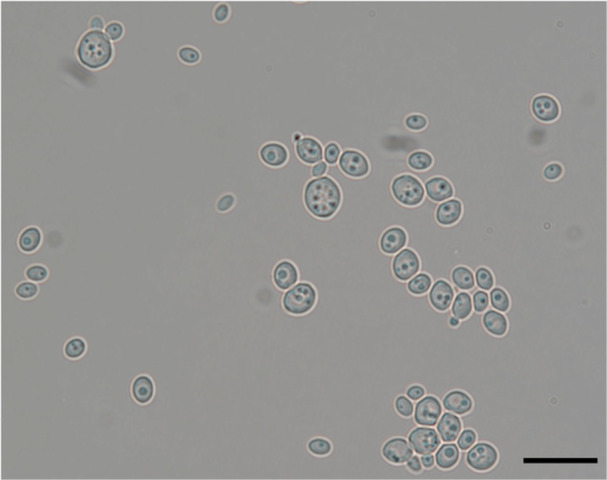
Cell morphology of *Lachancea rosae* sp. nov. (yHQL494) grown on YPDA at 25°C. Bars = 5 μm.

*Growth in 2% glucose‐yeast extract peptone broth and agar*: After 3 days of aerobic growth at 24°C on YPDA the colony is butyrous, tannish‐white, smooth with an entire border, and dull (Figure 3A). The cells are spherical to ellipsoidal, 2−5 × 2.5−6 μm and occur singly or in pairs (Figure 3B). No sexual reproduction was observed.

*Sexual reproduction*: No sexual structures (asci or ascospores) are observed, so it is designated f.a. for forma asexualis (Lachance 2012).

*Fermentation*: While maltose is fermented well, glucose and sucrose are weakly fermented; galactose, lactose, raffinose, and xylose are not fermented (Table 1).

**Table 1:** Physiological characteristics of yHQL494 and its closest relatives, *Lachancea meyerii* and *Lachancea lanzarotensis* (Kurtzman et al. 2011; Opulente et al. 2024). The symbols in the table represent strong growth (+), no growth (−), weak growth (w), delayed growth (d), variable growth (v), and where the data was unavailable (NA) (Kurtzman et al. 2011).

		yHQL494	*L. meyersii*	*L. lanzarotensis*
**Carbon Assimilation**	Glucose	+	+	+
	Mannose	+	NA	NA
	Fructose	d	NA	NA
	Inulin	+	v	—
	Sucrose	+	+	+
	Raffinose	+	+	+
	Melibiose	+	—	—
	Galactose	d	+	v
	Lactose	—	—	—
	Trehalose	+	+	+
	Maltose	+	+	+
	Melezitose	+	+	+
	Methyl‐alpha‐D‐glucoside	+	+	+
	Cellobiose	—	—	—
	Salicin	w/d	—	—
	L‐Sorbose	+	v	—
	Rhamnose	—	—	—
	Xylose	w	—	—
	L‐Arabinose	—	—	—
	D‐Arabinose	—	—	—
	Ribose	—	—	—
	Methanol	—	—	—
	Ethanol	+	+	+
	Glycerol	d	+	v
	Adonitol (syn. Ribitol)	d	v	—
	Erythritol	—	—	—
	Xylitol	—	—	v
	Galactitol	—	—	—
	Mannitol	d	+	+
	Sorbitol	+	+	+
	Myo‐inositol	—	—	—
	DL‐Lactate	—	—	—
	Succinate	—	—	—
	Citrate	—	—	—
	Gluconate	—	v	+
	Glucosamine	—	—	—
	N‐Acetyl‐D‐glucosamine	—	—	NA
	Hexadecane	—	—	NA
**Nitrogen**	Creatinine	—	—	NA
	Nitrate	—	—	—
	Nitrite	—	—	—
	Lysine	+	+	—
	Allantoin	+	NA	NA
**Fermentation**	Glucose	+	+	+
	Galactose	—	—	v
	Sucrose	+	+	+
	Maltose	+	+	+
	Lactose	—	—	—
	Raffinose	—	+	NA
	Xylose	—	NA	NA
**Temperature**	22°C	+	+	+
	30°C	+	+	+
	37°C	—	—	—
	42°C	—	—	—
	45°C	—	—	—
	50°C	—	—	—

*Assimilation of carbon compounds*: Growth is observed in glucose, sorbitol, maltose, ethanol, and sucrose media. Delayed growth is observed in mannitol, glycerol, adonitol (syn. ribitol), fructose, and galactose media. Weak growth is observed in d‐xylose and citrate media, and no growth is observed in ribose, succinate, DL‐lactate, cellobiose, d‐arabinose, myo‐inositol, l‐rhamnose, lactose, xylitol, glucosamine, and gluconate (Table 1).

*Assimilation of nitrogen compounds*: Growth occurs in allantoin. Weak growth is observed in lysine, while creatine, nitrate, and nitrite show no growth (Table 1).

*Temperature growth*: Growth is observed at room temperature and 30°C. No growth is observed at 37°C.

*Holotype:* Strain yHQL494^T^ was isolated from the hip of the rose *R. californica* in Castle Crags State Park, California (GPS: 41.152729, −122.311318) and is cryopreserved in a metabolically inactive state in the yH strain collection at the University of Wisconsin‐Madison. Ex‐type strains are CBS 18,575^T^ and NRRL Y‐64858^T^.

*Etymology:* The epithet ro.sae L. fem. gen. n., *rosae* refers to *Rosa*, the genus of the host plant, *R. californica*, from which the type strain was isolated.

*Genome sequence data*: Whole genome analyses were performed using the publicly available genome JAJMGS000000000 and short read data SRR16988996 (Opulente et al. 2024).

## 7 Conclusions

The availability of whole genome sequencing data for multiple yeast species and advances in sequencing technologies and bioinformatic tools have greatly aided in the exploration of yeast biodiversity. In this study, we propose the name *L. rosae* for a new species, isolated from the hip bud of *R. californica* in Castle Crags State Park. Phylogenetic analyses place it within the genus *Lachancea*. While physiological and genomic data supported some genotype‐phenotype associations, others revealed mismatches, highlighting the complexity of metabolic pathways. Taxogenomic studies like this one contribute to resolving such discrepancies and laying the groundwork for future work, including machine learning approaches, to uncover alternative mechanisms underlying yeast traits. Overall, this study adds to the growing collection of diverse yeasts and demonstrates the power of integrated approaches in discovering and characterizing novel species.

## Author Contributions

Dana A. Opulente, Quinn K. Langdon, and Chris Todd Hittinger conceived and designed the study. Yakendra Bajgain, Cara M. Krien, Anthony Pasles, Martin Jarzyna, Kelly V. Buh, Max A. B. Haase, Dana A. Opulente, and Quinn K. Langdon performed experiments. Yakendra Bajgain, Dana A. Opulente, Quinn K. Langdon, John F. Wolters, Martin Jarzyna, Kelly V. Buh, Max A. B. Haase, and Marizeth Groenewald analyzed data. Dana A. Opulente, Quinn K. Langdon, and Chris Todd Hittinger provided mentorship. Quinn K. Langdon and Chris Todd Hittinger secured funding. Yakendra Bajgain, Dana A. Opulente, and Chris Todd Hittinger wrote the paper with editorial input and approval from all authors.

## Conflicts of Interest

The authors declare no conflicts of interest.

## Supporting information


**Figure S1:** A) Neighbor‐Joining tree for strains of the 11 described *Lachancea* species and *Lachancea rosae* sp. nov. (yHQL494) based on the D1/D2 region of the LSU rRNA gene (Table S2). Bootstrap values (n = 1000) are indicated by numbers at the nodes. B) Neighbor‐Joining tree for strains of the 11 described *Lachancea* species and *Lachancea rosae* sp. nov. (yHQL494) based on the ITS region (Table S2). Bootstrap values (n = 1000) are indicated by numbers at the nodes.

**Table S1:** Isolation information and ITS and D1/D2 sequences for yHQL494.
**Table S2:** Strain information and accession numbers for ITS and D1/D2 sequences used in phylogenetic analyses (Figure 1A, Figure S1A – B).
**Table S3:** Comparison of LSU and ITS sequences between yHQL494 and its closest relatives, *Lachancea meyerii*, *Lachancea lanzarotensi, Lachancea nothofagi*, and *Lachancea dasiensis*.
**Table S4:** Newick tree of *Lachancea* species for the D1/D2 region of the LSU rRNA gene.
**Table S5:** Pairwise average nucleotide index (ANI) values.
**Table S6:** Results from TBLASTX for all genes in Figure 2.

## Data Availability

All gene and genome sequence data have been deposited in GenBank under the accession numbers described above. All other data are contained within the manuscript and its supplements.
