# Supplementary figures and images for: Taxogenomic Analysis of a Novel Yeast Species, Lachancea rosae Sp. Nov. F.A., Isolated From the Wild Rose Rosa californica
Source: Yeast. 2025 Sep 1;42(11):213–21. doi: 10.1002/yea.70000 (PMC12587028; doi:10.1002/yea.70000)

A

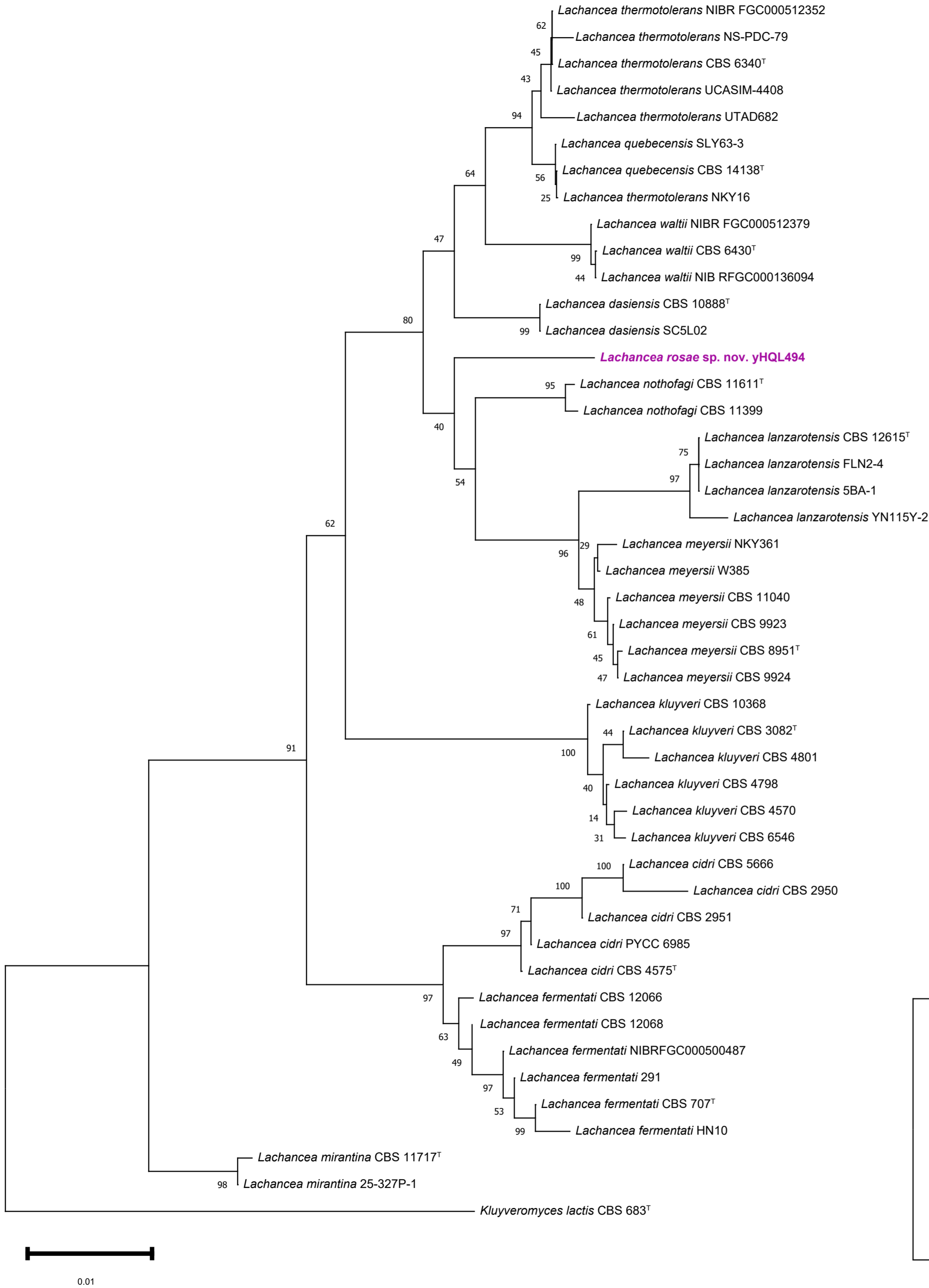

B

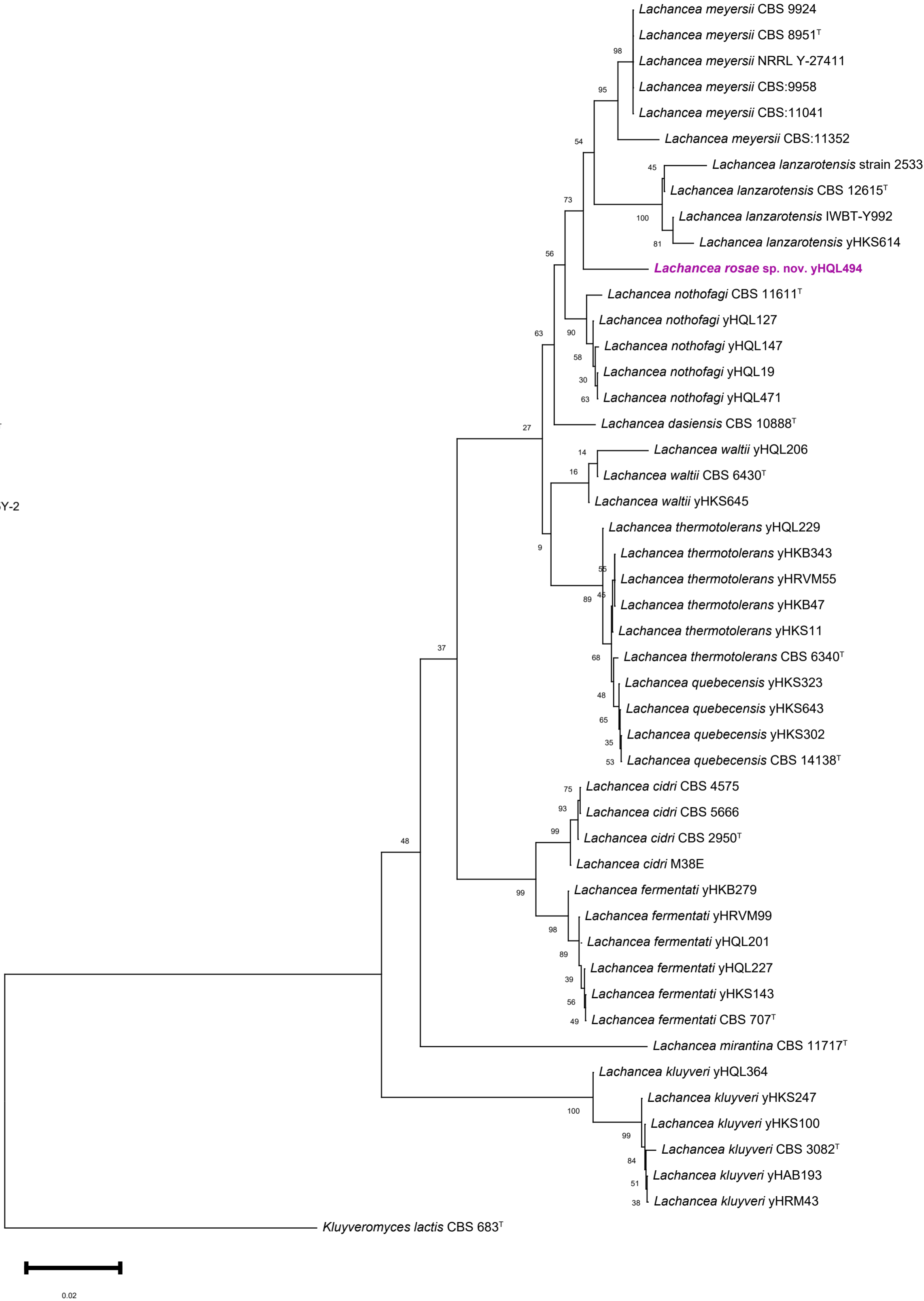

Supplement: Supplementary file 1 — Figure S1: A) Neighbor‐Joining tree for strains of the 11 described Lachancea species and Lachancea rosae sp. nov. (yHQL494) based on the D1/D2 region of the LSU rRNA gene (Table S2). Bootstrap values (n = 1000) are indicated by numbers at the nodes. B) Neighbor‐Joining tree for strains of the 11 described Lachancea species and Lachancea rosae sp. nov. (yHQL494) based on the ITS region (Table S2). Bootstrap values (n = 1000) are indicated by numbers at the nodes. [file YEA-42-213-s002.pdf]
